# Predicting miRNA-disease associations based on PPMI and attention network

**DOI:** 10.1186/s12859-023-05152-z

**Published:** 2023-03-23

**Authors:** Xuping Xie, Yan Wang, Kai He, Nan Sheng

**Affiliations:** 1 Key Laboratory of Symbol Computation and Knowledge Engineering of Ministry of Education, College of Computer Science and Technology, Jilin University, Changchun, China; 2 School of Artificial Intelligence, Jilin University, Changchun, China

**Keywords:** MiRNA-disease association prediction, PPMI, Attention network, Deep learning

## Abstract

**Background:**

With the development of biotechnology and the accumulation of theories, many studies have found that microRNAs (miRNAs) play an important role in various diseases. Uncovering the potential associations between miRNAs and diseases is helpful to better understand the pathogenesis of complex diseases. However, traditional biological experiments are expensive and time-consuming. Therefore, it is necessary to develop more efficient computational methods for exploring underlying disease-related miRNAs.

**Results:**

In this paper, we present a new computational method based on positive point-wise mutual information (PPMI) and attention network to predict miRNA-disease associations (MDAs), called PATMDA. Firstly, we construct the heterogeneous MDA network and multiple similarity networks of miRNAs and diseases. Secondly, we respectively perform random walk with restart and PPMI on different similarity network views to get multi-order proximity features and then obtain high-order proximity representations of miRNAs and diseases by applying the convolutional neural network to fuse the learned proximity features. Then, we design an attention network with neural aggregation to integrate the representations of a node and its heterogeneous neighbor nodes according to the MDA network. Finally, an inner product decoder is adopted to calculate the relationship scores between miRNAs and diseases.

**Conclusions:**

PATMDA achieves superior performance over the six state-of-the-art methods with the area under the receiver operating characteristic curve of 0.933 and 0.946 on the HMDD v2.0 and HMDD v3.2 datasets, respectively. The case studies further demonstrate the validity of PATMDA for discovering novel disease-associated miRNAs.

## Background

MicroRNAs (miRNAs) are a class of small endogenous non-coding RNAs that do not encode proteins. MiRNAs are approximately 22nt in length and bind to the $3^{\prime }$ untranslated region of target mRNAs mainly through sequence-specific base pairing, which in turn participates in the regulation of target mRNA expression at the post-transcriptional level [1–4]. More and more studies have shown that mutation or abnormal expression of miRNAs is often linked to the development and progression of complex human diseases such as cancer [5, 6]. For example, miR-143 and -145 consistently show reduced stable levels of mature miRNAs in adenoma and carcinoma stages of colorectal cancer [7]. In lung cancer, high hsa-mir-155 and low hsa-let-7a-2 expression are associated with poor survival and they may be potential prognostic markers [8]. Therefore, it is necessary to reveal more underlying associations between miRNAs and diseases for the sake of understanding the pathogenesis and developing personalized therapies.

Experimental methods such as qPT-PCR [9], northern blotting [10], and microarray profiling [11] have been used to predict novel miRNAs associated with diseases. Although experimental methods have highly accurate results, they usually require a relatively large time and economic investment, which is inefficient. Therefore, to facilitate the discovery of potential disease-related miRNAs, computational methods are developed, which can be classified into three main types, namely similarity-based methods, machine learning-based methods, and deep learning-based methods.

For similarity-based methods, they are based on the assumption that functionally similar miRNAs tend to correlate with similar diseases and vice versa. Jiang et al. [12] put forward the first computational method for miRNA-disease association (MDA) prediction, which uses hypergeometric probability distributions to explore disease-related miRNAs. However, it was oversimplified by using the Boolean network to reflect the associations between diseases or between miRNAs, which may result in loss of information. Subsequently, HDMP [13] was proposed to assess the functional similarity between two miRNAs based on known MDAs and semantic similarity of diseases and to predict the MDA scores based on weighted k most similar neighbors. This approach overcomes the drawback of the Boolean network by calculating the similarities of miRNAs and diseases, but they both only consider the direct neighbor information (local information) of the network and ignore the global information in the network. Further, Chen et al. [14] devised a method based on global network similarity, which identifies potential disease-associated miRNAs by performing random walk with restart (RWR) on the disease similarity network. And Shi et al. [15] combined a protein-protein interaction (PPI) network and utilized RWR to predict underlying associations between miRNAs and diseases, which takes advantage of the association information between miRNAs or diseases and genes. Although these methods are gradually improving the performance of MDA prediction, they are difficult in predicting miRNAs associated with new diseases that have no known relevant miRNAs. To address this problem, Chen et al. [16] proposed to employ regularized least squares to uncover novel MDAs, which is a semi-supervised and global approach. Although some similarity-based methods attempt to improve the performance of identifying new MDAs, including the potential associations for new diseases and new miRNAs [17–19], they are susceptible to the quality of the networks constructed, such as different similarity calculation methods may yield different results.

Machine learning-based methods are another class of computational methods that are often used for predicting MDAs. For example, EGBMMDA [20] employed the extreme gradient boosting machine to obtain the probability scores of relationships between miRNAs and diseases, and it was the first decision tree learning-based model for inferring candidate miRNAs. Chen et al. [21] designed a computational model that uses a filter-based method to select important features and employed random forest to discover disease-associated miRNAs. In addition, Zhang et al. [22] proposed a graph regularized generalized matrix factorization method to screen novel miRNAs that are related to diseases, which takes into account the neighborhood information of each node. NCMCMDA [23] combined neighborhood constraints with matrix completion to reconstruct the relationship matrix between miRNAs and diseases. However, these models above extract and fuse feature at shallow levels, which are unable to learn complex latent associations from multi-source data.

In the last few years, deep learning has achieved satisfactory results in many domains, and as a result, using deep learning to predict molecular associations has become a hot topic. For example, SAEMDA [24] pre-trained the stacked auto-encoder (SAE) with all MDA pairs, and then fine-tuned the SAE with an equal number of known and unobserved MDA pairs to determine potential disease-related miRNAs. Although deep learning-based methods [25–27] have achieved good performance on intermolecular relationship prediction, some of them ignore the information interaction between nodes on the heterogeneous network composed of different biological entities. Recently, to exploit known molecular relationship pairs for information fusion between different types of nodes, Long et al. [28] used graph attention networks with talking-heads to learn embeddings of microbes and diseases based on the microbe-disease association network, and GAEMDA [29] is a new graph auto-encoder method that aggregates the neighborhood information of nodes based on known MDAs via the aggregator function and multi-layer perceptron, which achieves heterogeneous information fusion. Nevertheless, most of these models only consider the first-order proximity of the nodes in simple integrated similarity networks, while ignoring the multi-hop neighborhood information in different similarity networks. Some studies [25, 30, 31] have shown that high-order neighborhood information in networks is important for learning embedding representations of nodes on homogeneous/heterogeneous networks. Therefore, to learn high-order proximity representations of nodes from different similarity networks and efficiently fuse information of different types of nodes, we develop a new end-to-end computational approach based on positive point-wise mutual information (PPMI) and attention network for predicting MDAs, called PATMDA. Specifically, our main contributions are summarized as follows:

- We construct the MDA network and multiple miRNA and disease similarity networks, which are based on disease semantic similarity, miRNA functional similarity, and Gaussian interaction profile (GIP) kernel similarity for miRNAs and diseases.
- To learn global structural information from the similarity network views, RWR and PPMI are utilized to obtain multi-order proximity features. Furthermore, we combine high-order proximity representations got by exploiting convolutional neural network (CNN) and first-order proximity representations including direct neighbor information.
- To efficiently integrating structural features of different types of nodes, we design an attention network with neural aggregation, which learns the final representations of miRNA and disease nodes by fusing the representations of nodes and their heterogeneous neighbors based on the MDA network.
- Our experimental results show PATMDA outperforms baseline methods in exploring novel MDAs.

## Results and discussion

### Datasets

In this work, we obtain human MDAs from HMDD v2.0 [32] that are confirmed by experimental evidence in the literature, including 5430 known MDAs among 495 miRNAs and 383 diseases. In addition, the newest version HMDD v3.2 [33] is used to further validate the performance of the model, where as in [25], 12 446 observed MDAs involving 853 miRNAs and 591 diseases are extracted. And directed acyclic graphs (DAGs) about the semantic trees of diseases are downloaded from the medical subject heading (MeSH) (https://www.nlm.nih.gov/mesh/).

### Experimental setup

The PATMDA model is implemented based on the Pytorch framework. The Xavier normal distribution is employed for the initialization of the transformation matrices. For the hyperparameters of the model, we set the number of RWR transition steps *K* as 3, the number of CNN filters $C_{out}$ for miRNA and disease as 256, the dimensionality of the transformed feature $f_{tran}$ as 256, attentional heads’ number *L* as 2, and the learning rate as 0.0001.

In addition, common classification evaluation metrics are used to evaluate the performance of PAMDA for predicting MDAs, which includes area under the receiver operating characteristic (ROC) curve (AUC), area under the precision/recall (P–R) curve (AUPR), area under the TruePositiveRate@k (TPR@k) curve (AUTPR@k), accuracy (Acc.), precision (Prec.), recall and F1-score. And we plot the ROC curve, P–R curve, and TPR@k curve to evaluate the PATMDA performance, where TPR@k curve depicts the proportion of positive samples predicted correctly in the top k to all positive samples under different k values [34]. It is worth noting that the calculation of the evaluation metrics, such as precision, would involve the existence of negative samples or making some assumptions about unknown samples. Since there are no proven uncorrelated miRNA-disease pairs, we make the assumption that MDA pairs that are not verified are considered negative samples. Further, in the experiment, we take all known MDA pairs as positive samples, and randomly select an equal number of samples from unconfirmed MDA pairs as negative samples. We use 5-fold cross-validation (5-CV) to evaluate the PATMDA model. Specifically, all samples are randomly divided into 5 equal parts, and in turn, each part is treated as the test set while the others are applied for training. In each round of 5-CV, the GIP kernel similarity of miRNAs and diseases is recalculated based on the training set.

### Performance evaluation

**Fig. 1:**
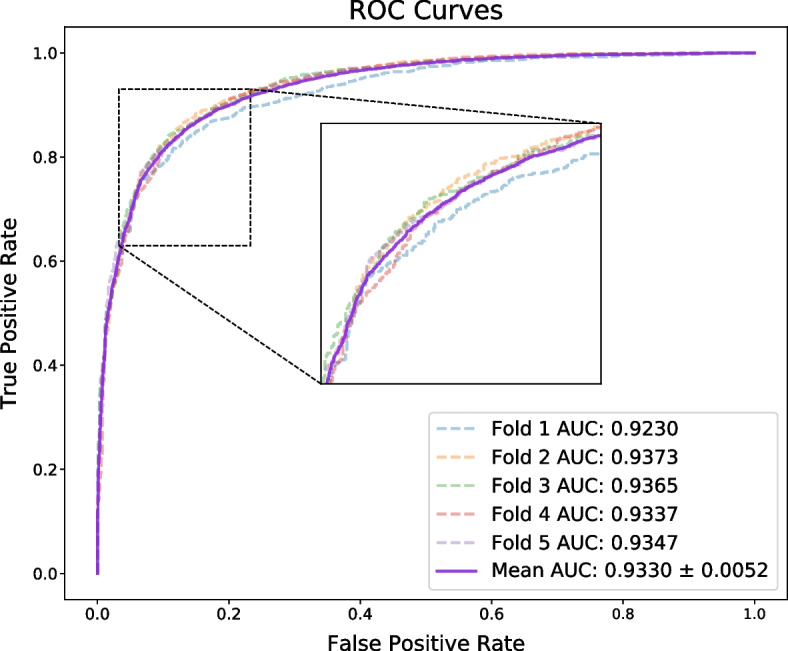
ROC curves of PATMDA under 5-CV based on HMDD v2.0

**Fig. 2:**
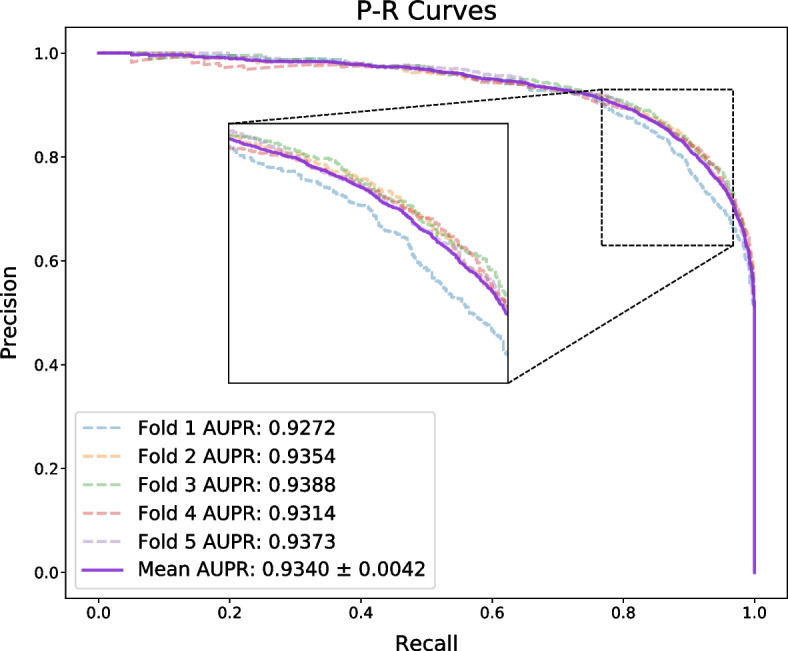
P–R curves of PATMDA under 5-CV based on HMDD v2.0

**Fig. 3:**
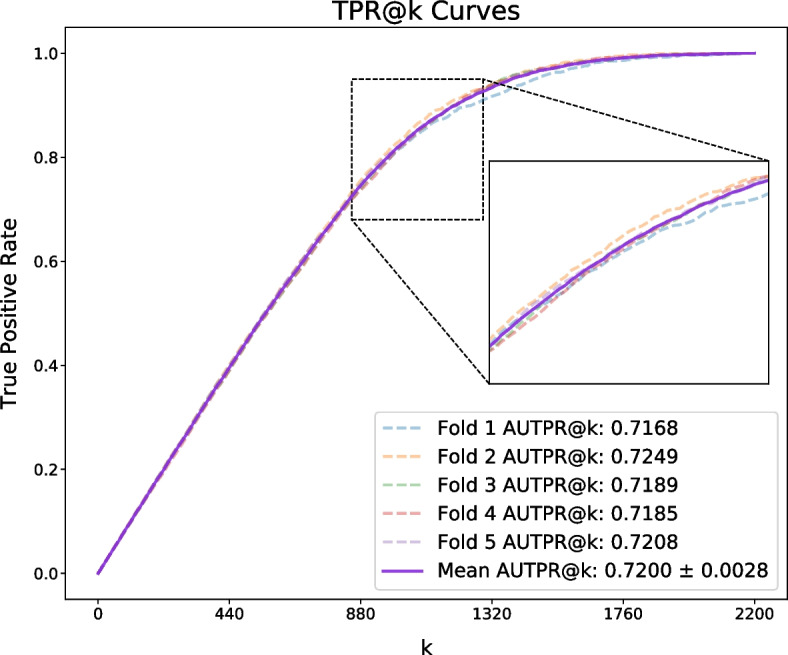
TPR@k curves of PATMDA under 5-CV based on HMDD v2.0

Here, we evaluate the performance of PATMDA on HMDD v2.0 dataset using 5-CV. As shown in Table 1, PATMDA obtains mean Acc. of 85.78$\%$, Prec. of 85.27$\%$, recall of 86.53$\%$, and F1-score of 85.88$\%$. In addition, Figs. 1, 2 and 3 show the ROC, P–R and TPR@k curves of the PATMDA model, respectively. We are able to see that PATMDA obtains mean AUTPR@k of 72$\%$, achieves mean AUC of 93.3$\%$, which is the mean of 92.3$\%$, 93.73$\%$, 93.65$\%$, 93.37$\%$, 93.47$\%$, and obtains average AUPR of 93.4$\%$, which is the average of 92.72$\%$, 93.54$\%$, 93.88$\%$, 93.14$\%$, 93.73$\%$.

**Table 1:** The results of PATMDA based on HMDD v2.0

Testing set	Acc. (%)	Prec. (%)	Recall (%)	F1-score (%)
1	84.71	84.02	85.42	84.71
2	86.33	84.57	88.43	86.46
3	86.1	86.47	86	86.24
4	85.96	86.31	85.75	86.03
5	85.82	84.98	87.02	85.99
Mean	$85.78\pm 0.56$	$85.27\pm 0.97$	$86.53 \pm 1.09$	$85.88 \pm 0.61$

### Comparison with state-of-the-art methods

To further evaluate the performance of our proposed model, we compare the PATMDA model with six state-of-the-art computational models for predicting MDAs using 5-CV on HMDD v2.0 and HMDD v3.2 datasets, including MDHGI [35], ABMDA [36], NIMCGCN [27], DANE-MDA [37], SAEMDA [24] and MINIMDA [31]. MDHGI is a method for identifying potential disease-related miRNAs by using matrix decomposition and heterogeneous graph inference [35]. ABMDA is an adaptive boosting-based method for uncovering underlying associations between miRNAs and diseases [36]. NIMCGCN is a computational method that combines neural inductive matrix completion and graph convolutional network to predict MDAs [27], and DANE-MDA reveals latent MDAs based on deep attributed network embedding [37]. SAEMDA is a stacked autoencoder-based approach for prioritizing disease-related miRNAs [24], and MINIMDA discovers potential relationships between miRNAs and diseases by integrating mixed neighborhood information in multimodal networks [31]. For a fair comparison, we adopt the default parameters of the baseline models provided by the authors to obtain their AUC and AUPR.

**Fig. 4:**
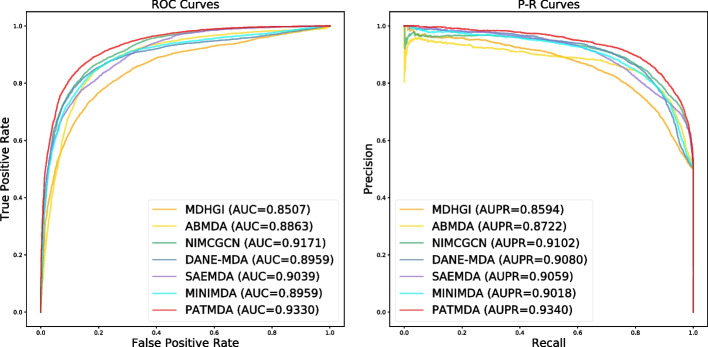
ROC curves and P–R curves of PATMDA with all comparison methods under 5-CV on HMDD v2.0 dataset

**Fig. 5:**
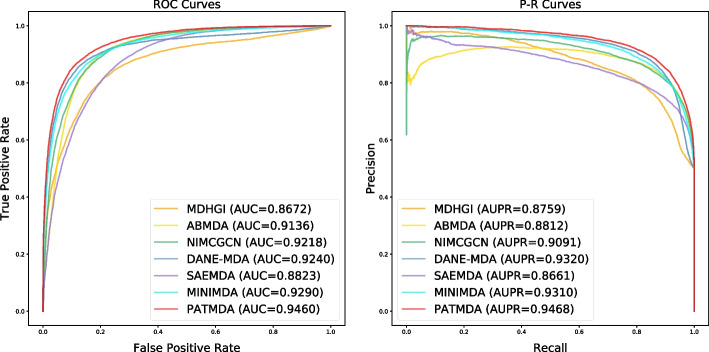
ROC curves and P–R curves of PATMDA with all comparison methods under 5-CV on HMDD v3.2 dataset

As shown in Figs. 4 and 5, PATMDA achieves competitive performance on both datasets. For HMDD v2.0 dataset, PATMDA achieves the highest AUC, AUPR of 93.3%, 93.4%, which may be due to that PATMDA fuses multi-order proximity representations from multiple similarity network views via CNN and aggregates heterogeneous structural information of miRNA and disease nodes via attention network with neural aggregation. Compared with MDHGI, ABMDA, and SAEMDA, PATMDA takes into account the high-order proximity representations of nodes in different similarity networks and enhances the information interaction between heterogeneous nodes, instead of using only direct neighbor information from the integrated similarity network as in these comparison methods. Although DANE-MDA considers the interaction between the association network structure and similarity (attribute) information of miRNAs and diseases captured from the diverse degrees of proximity, its performance is not as good as that of PATMDA, which suggests that the design of our model is more reasonable. In addition, although NIMCGCN utilizes high-order information from the integrated similarity network, its performance is not as good as that of PATMDA, which may be due to the fact that NIMCGCN ignores the information fusion between miRNA and disease nodes. Though MINIMDA considers high-order information of nodes and information fusion between heterogeneous nodes, its performance is still worse than that of PATMDA, which may be because PATMDA can better capture and fuse information of nodes in different networks and has better representation ability. Furthermore, Fig. 5 shows that PATMDA achieves the highest AUC, AUPR of 94.6%, 94.68% on HMDD v3.2 dataset. In conclusion, the experimental results show that PATMDA is effective in exploring potential disease-related miRNAs.

### Ablation experiments

**Fig. 6:**
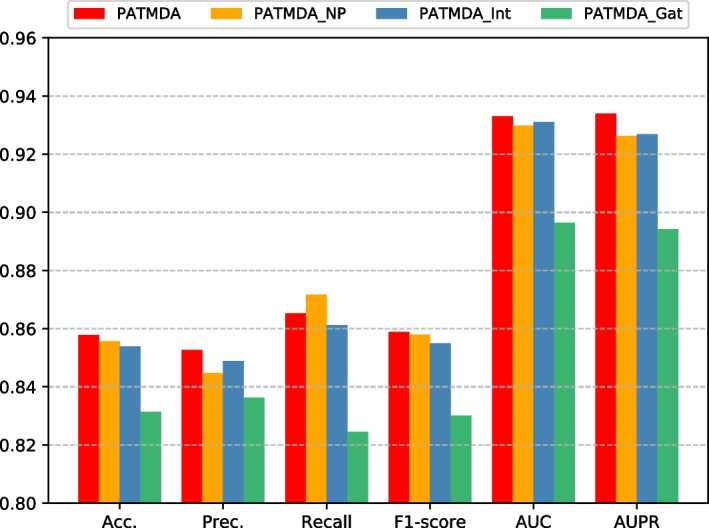
The performance of PATMDA and its variants under 5-CV on HMDD v2.0 dataset

We use an attention network with neural aggregation to efficiently aggregate information of the nodes and their heterogeneous neighbors, and obtain structural features of nodes containing first-order and high-order proximity from multiple similarity network views. To analyze the importance of the main components of our model, we design three variants of PATMDA (PATMDA_NP, PATMDA_Int, PATMDA_Gat) as comparison methods. PATMDA_NP means that we do not consider high-order proximity representations of nodes. Besides, like most methods, PATMDA_Int uses GIP kernel similarity to fill in missing values for another similarity, instead of considering each similarity view separately. PATMDA_Gat uses the standard graph attention network (GAT) [38] to replace the attention network with neural aggregation module, which does not effectively consider the importance of the nodes themselves. Figure 6 shows the evaluation results of PATMDA and its variant models under 5-CV on HMDD v2.0 dataset, except that the recall of PATMDA is lower than that of PATMDA_NP, all other indicators are significantly higher than the variant models. For PATMDA_NP and PATMDA, after combining high-order proximity information, this model can obtain more structural information than only considering first-order proximity information. This result shows that high-order proximity and first-order proximity representations contain different structural features, which means that high-order information can be used as a complement to first-order information. For PATMDA_Int and PATMDA, it is more beneficial to consider the structural information in each similarity network separately to extract the feature representations of miRNAs and diseases. For PATMDA_Gat and PATMDA, compared with only considering the information of its neighbor nodes, after using neural aggregation to enhance the information interaction between the node and its neighbor nodes, the model obtains more informative representations, which proves that the information of the node itself is also very important.

### Case studies

To further validate the ability of the PATMDA model to discover potential disease-related miRNAs in practical applications, we conduct two different types of case studies based on the HMDD v2.0 dataset, and the dbDEMC [39] and HMDD v3.2 [33] datasets are utilized to identify the top 20 new associations between miRNAs and diseases.

In the first case study, we focus on detecting novel MDAs. Specifically, for each specific disease, we use all known MDA pairs and an equal number of association pairs randomly selected from unknown MDA pairs except those associated with the disease to train the PATMDA model, and further predict probability scores for unknown relationship pairs related to the disease. Furthermore, we rank potential miRNAs in descending order based on the predicted scores. We establish the case study for three diseases, including lymphoma, prostate neoplasms, and esophageal neoplasms. Tables 2, 3 and 4 respectively show the top 20 miRNA candidates associated with three diseases, which can be identified and validated by dbDEMC or HMDD v3.2 datasets.

In the second case study, we attempt to validate the usability of the PATMDA model for new diseases without observed associated miRNAs, where we take the relationship pairs between a specific disease and all miRNAs as the test set, and use the remaining known relationship pairs and a randomly selected equal number of unobserved relationship pairs related to other diseases as the training set. Similarly, we prioritize the top 20 underlying miRNAs according to the relationship scores predicted by PATMDA. Here we predict the associations between miRNAs and breast neoplasms, one of the most common malignancies in women. As shown in Table 5, the top 20 predicted breast neoplasm-related miRNAs are all confirmed by dbDEMC, and 12 of them are also verified by HMDD v3.2.

The results of the above case studies demonstrate that PATMMDA has good performance in screening latent MDAs and miRNA candidates associated with new diseases.

**Table 2:** The top 20 miRNAs associated with lymphoma

Rank	miRNA	Evidence	Rank	miRNA	Evidence
1	hsa-mir-125b	dbDEMC	11	hsa-let-7b	dbDEMC
2	hsa-mir-34a	dbDEMC	12	hsa-let-7g	dbDEMC
3	hsa-mir-181b	dbDEMC	13	hsa-let-7a	dbDEMC
4	hsa-mir-221	dbDEMC; HMDDv3.2	14	hsa-mir-34c	dbDEMC
5	hsa-mir-106b	dbDEMC	15	hsa-let-7i	dbDEMC
6	hsa-mir-223	dbDEMC	16	hsa-mir-222	dbDEMC; HMDDv3.2
7	hsa-mir-145	dbDEMC	17	hsa-let-7d	dbDEMC; HMDDv3.2
8	hsa-mir-29b	dbDEMC; HMDDv3.2	18	hsa-mir-143	dbDEMC; HMDDv3.2
9	hsa-mir-29a	dbDEMC	19	hsa-let-7e	dbDEMC
10	hsa-let-7c	dbDEMC	20	hsa-mir-146b	dbDEMC

**Table 3:** The top 20 miRNAs associated with prostate neoplasms

Rank	miRNA	Evidence	Rank	miRNA	Evidence
1	hsa-mir-21	dbDEMC; HMDDv3.2	11	hsa-mir-106b	dbDEMC; HMDDv3.2
2	hsa-mir-155	dbDEMC; HMDDv3.2	12	hsa-let-7b	dbDEMC; HMDDv3.2
3	hsa-mir-34a	dbDEMC; HMDDv3.2	13	hsa-mir-182	dbDEMC; HMDDv3.2
4	hsa-mir-146a	dbDEMC; HMDDv3.2	14	hsa-mir-15a	dbDEMC; HMDDv3.2
5	hsa-mir-126	dbDEMC; HMDDv3.2	15	hsa-mir-29a	dbDEMC; HMDDv3.2
6	hsa-mir-16	dbDEMC; HMDDv3.2	16	hsa-mir-34c	dbDEMC; HMDDv3.2
7	hsa-mir-199a	dbDEMC; HMDDv3.2	17	hsa-mir-200b	dbDEMC; HMDDv3.2
8	hsa-mir-29b	dbDEMC; HMDDv3.2	18	hsa-mir-223	dbDEMC; HMDDv3.2
9	hsa-mir-15b	dbDEMC; HMDDv3.2	19	hsa-mir-222	dbDEMC; HMDDv3.2
10	hsa-mir-143	dbDEMC; HMDDv3.2	20	hsa-mir-221	dbDEMC; HMDDv3.2

**Table 4:** The top 20 miRNAs associated with esophageal neoplasms

Rank	miRNA	Evidence	Rank	miRNA	Evidence
1	hsa-mir-125b	dbDEMC; HMDDv3.2	11	hsa-let-7g	dbDEMC; HMDDv3.2
2	hsa-let-7d	dbDEMC	12	hsa-let-7e	dbDEMC
3	hsa-mir-200b	dbDEMC	13	hsa-mir-181a	dbDEMC
4	hsa-mir-16	dbDEMC	14	hsa-mir-146b	dbDEMC; HMDDv3.2
5	hsa-mir-9	dbDEMC; HMDDv3.2	15	hsa-let-7i	dbDEMC; HMDDv3.2
6	hsa-mir-17	dbDEMC	16	hsa-mir-181b	dbDEMC
7	hsa-mir-222	dbDEMC	17	hsa-mir-125a	dbDEMC
8	hsa-let-7f	dbDEMC	18	hsa-mir-221	dbDEMC; HMDDv3.2
9	hsa-mir-1	dbDEMC	19	hsa-mir-7	dbDEMC
10	hsa-mir-29a	dbDEMC	20	hsa-mir-29b	dbDEMC

**Table 5:** The top 20 miRNAs associated with breast neoplasms. The miRNAs related to breast neoplasms are deleted before training the PATMDA model

Rank	miRNA	Evidence	Rank	miRNA	Evidence
1	hsa-mir-1207	dbDEMC; HMDDv3.2	11	hsa-mir-641	dbDEMC
2	hsa-mir-21	dbDEMC; HMDDv3.2	12	hsa-mir-146a	dbDEMC; HMDDv3.2
3	hsa-mir-659	dbDEMC	13	hsa-mir-545	dbDEMC
4	hsa-mir-451	dbDEMC; HMDDv3.2	14	hsa-mir-4792	dbDEMC
5	hsa-mir-189	dbDEMC	15	hsa-mir-125b	dbDEMC; HMDDv3.2
6	hsa-mir-155	dbDEMC; HMDDv3.2	16	hsa-mir-298	dbDEMC; HMDDv3.2
7	hsa-mir-941	dbDEMC	17	hsa-mir-1827	dbDEMC
8	hsa-mir-548c	dbDEMC; HMDDv3.2	18	hsa-mir-122	dbDEMC; HMDDv3.2
9	hsa-mir-922	dbDEMC; HMDDv3.2	19	hsa-mir-34a	dbDEMC; HMDDv3.2
10	hsa-mir-1287	dbDEMC	20	hsa-mir-145	dbDEMC; HMDDv3.2

## Conclusion

In this paper, we propose a novel computational approach named PATMDA, which combines PPMI and attention network with neural aggregation to identify unobserved associations between miRNAs and diseases. PATMDA not only considers the first-order neighbor information in different similarity network views, but also efficiently extracts high-order neighbor information from similarity views by using PPMI and CNN. To obtain more informative representations, we use an attention network with neural aggregation to integrate the structural information of heterogeneous nodes according to the MDA network. Comprehensive experiments show that our proposed PATMDA model is reliable and efficient in retrieving potential miRNA candidates for diseases, which may contribute to guiding biological experiments.

However, there are still some limitations that need to be further investigated in the future. First, although we consider the topology features from multiple similarity network views, how to maintain the consistency and complementarity of features learned from different similarity views is a topic worthy of future research. Second, in the similarity calculation of diseases and miRNAs, we hope to introduce more information to discover disease-related miRNAs, such as miRNA sequence similarity and gene-based functional similarity of miRNAs and diseases may help in MDA prediction. In conclusion, more and more biological data sources provide convenience for predicting MDAs, but how to more effectively and rationally apply information from different data sources to improve the performance of methods for inferring potential miRNAs for diseases requires further exploration. In addition, we will also try to use PATMDA to identify non-coding RNAs such as lncRNAs and circRNAs that are related to diseases, and further design a more general method for predicting the relationships between non-coding RNAs and diseases.

## Methods

**Fig. 7:**
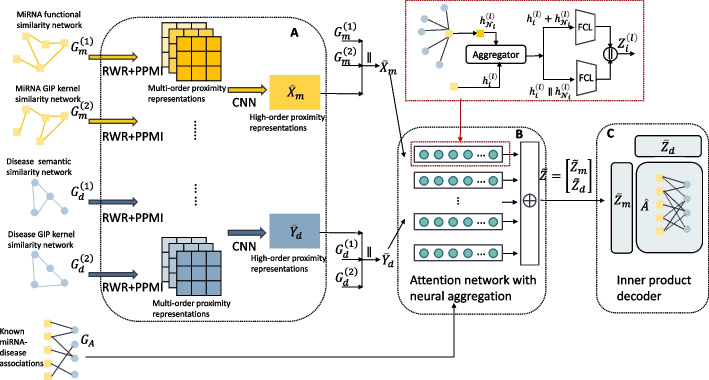
Overview of PATMDA model architecture. **a** Learning high-order proximity representations. **b** Fusing structural information of heterogeneous nodes. **c** Association prediction for miRNAs and diseases

In this work, we put forward a deep learning model based on PPMI and attention network for MDA prediction. As shown in Fig. 7, PATMDA mainly consists of the following parts: (i) RWR and PPMI are applied to various similarity network views to learn multi-order proximity representations, in turn, CNN is employed to obtain high-order proximity representations by fusing the learned multi-order proximity features; (ii) based on the combined first-order and high-order proximity representations, the information of the nodes and their heterogeneous neighbors is integrated by the attention network with neural aggregation to obtain the final embeddings; (iii) inner product decoder is used to predict the association probability scores between miRNAs and diseases.

### Human MDAs

Based on known human MDAs, we get an association matrix $A\in R^{nm*nd}$ between miRNAs and diseases, where *nm* and *nd* denote the number of miRNAs and diseases, respectively. When there is a verified relationship between miRNA *i* and disease *j*, $A_{ij}$ is equal to 1, otherwise, it is 0. Further, we construct the heterogeneous association network including miRNA and disease nodes based on the relationship matrix, whose adjacency matrix $G_{A}$ can be defined as follows:

1 $$\begin{aligned} \begin{aligned} G_{A} =\begin{bmatrix} 0 &{} A \\ A^{T} &{} 0 \end{bmatrix} . \end{aligned} \end{aligned}$$

### Similarity measures

#### Disease semantic similarity

Disease semantic similarity can be calculated based on MeSH descriptors [13], in which the relationships between diseases can be described by DAGs. Many studies [24, 27, 40] have used DAGs to generate disease semantic similarities (DSSs). There are two different approaches to calculating DSSs. DSS1 is obtained based on the assumption that two diseases are more similar to each other if they share more ancestral nodes in the DAGs. Further considering that the disease that appears in more (or less) DAGs is more common (or specific), DSS2 assigns different semantic contribution values to the diseases in the same layer of the DAG. We compute these two DSSs between diseases according to the previous method [13, 41] and obtain the adjacency matrix $G_d^{(1)}$ of the disease semantic similarity network by averaging them, which means the edge weight between two disease nodes is equal to their semantic similarity value.

#### MiRNA functional similarity

On the basis of the hypothesis that similar functional miRNAs tend to correlate with similar phenotypic diseases and vice versa, miRNA functional similarity can be calculated according to the method in a previous study [41]. That is, we are able to obtain miRNA functional similarity depending on the semantic similarity between miRNA-related disease sets. For fairness, for the HMDD v2.0 dataset, we acquire the miRNA functional similarity directly from https://www.cuilab.cn/files/images/cuilab/misim.zip as in many studies. Whereas for the HMDD v3.2 dataset, we generate the functional similarity between miRNAs following [41]. In turn, we obtain the adjacency matrix $G_m^{(1)}$ of the miRNA functional similarity network, where the similarity value between two miRNAs determines the edge weight between the two nodes.

#### GIP kernel similarity for diseases and miRNAs

Based on the assumption that similar diseases and miRNAs have analogous modes of interaction and non-interaction and vice versa [24], GIP kernel similarity can be used to measure the relationships between miRNAs and between diseases. miRNA GIP kernel similarity and disease GIP kernel similarity can be calculated by the approach in the previous study [42]. Similarly, based on the GIP kernel similarity of miRNAs and diseases, we can obtain the adjacency matrices $G_m^{(2)}$ and $G_d^{(2)}$ of their corresponding GIP similarity networks.

### Learning high-order proximity representations

As described in [43, 44], the edge weight between two nodes determines the first-order proximity between them, that is, the first-order proximity indicates the degree of similarity between two nodes. The high-order proximity between two nodes represents neighborhood similarity, meaning that two nodes are similar if they share similar neighbors. The high-order proximity includes the global structure information of the network, and the first-order proximity contains the local structure information of the network, which gives the direct neighbor information. Each similarity network view of miRNAs (diseases) gives first-order proximity information of miRNAs (diseases) from different perspectives. Next, we will introduce how to obtain the high-order proximity of nodes according to different similarity views.

#### Multi-order proximity representations by PPMI

Since only local structural information is contained in the similarity network, motivated by [45, 46], we adopt RWR [47] to capture the global topological information of different similarity network views. Specifically, every time, the random walk process will continue at probability $\alpha$ and will return to the initial node and restart the process at probability $1-\alpha$. We denote the *s*-th similarity view of miRNA and the *q*-th similarity view of disease as $G_m^{(s)}$ and $G_d^{(q)}$, respectively. For example, for the view *s* of miRNA, RWR can be represented as the following iterative process:

2 $$\begin{aligned} \begin{aligned} P_k^{(s)}=\alpha P_{k-1}^{(s)} \hat{G}_m^{(s)}+(1-\alpha ) P_0^{(s)} , \end{aligned} \end{aligned}$$

where $P_k^{(s)}$ denotes the transfer probability matrix after *k* steps in view *s*. $P_0^{(s)}$ is an identity matrix, and $\widehat{G}_m^{(s)}$ represents the one-step probability transition matrix got by employing row-wise normalization of the similarity weight matrix $G_m^{(s)}$. After the *K*-step, we obtain network structure information of view *s* from different orders of proximity with the probability transition matrices $\left\{ P_1^{(s)}, P_2^{(s)}, \cdots , P_K^{(s)}\right\}$, which characterizes the probability of co-occurrence of miRNA nodes on view *s* from different degrees.

Next, according to the multi-order proximity of miRNAs on view *s*, we obtain multi-order representations of miRNA nodes by computing a shifted PPMI matrix [48]. For the *k*-th step structure proximity $P_k^{(s)}$, the PPMI matrix is calculated as follow:

3 $$\begin{aligned} \begin{aligned} X_{k, i j}^{(s)}=\max \left( 0, \log _2\left( \frac{P_{k, i j}^{(s)} \sum _i \sum _j P_{k, i j}^{(s)}}{\sum _i P_{k, i j}^{(s)} \sum _j P_{k, i j}^{(s)}}\right) \right) , \end{aligned} \end{aligned}$$

where $k=1,2, \cdots , K$ and $X_{k, i j}^{(s)}$ is the *j*-th feature of miRNA *i* at the *k*-th step in view *s*. According to the view *s* of miRNAs, we get the multi-order representations of miRNAs as follows:

4 $$\begin{aligned} \begin{aligned} \left\{ X_1^{(s)}, X_2^{(s)}, \cdots , X_K^{(s)}\right\} . \end{aligned} \end{aligned}$$

Analogously, we can obtain multi-order feature representations of diseases on the *q*-th network view $G_d^{(q)}$:

5 $$\begin{aligned} \begin{aligned} \left\{ Y_1^{(q)}, Y_2^{(q)}, \cdots , Y_K^{(q)}\right\} , \end{aligned} \end{aligned}$$

where $Y_k^{(q)}$ is the *k*-th order representation of diseases obtained by using the RWR and PPMI to the normalized similarity weight matrix $G_d^{(q)}$ of view *q*, $k=1,2, \cdots , K$ and *K* denotes the total number of RWR transition steps.

As shown in Fig. 7a, by applying the RWR and PPMI to preprocess the multi-view of miRNAs and diseases, we can get the representations of miRNAs and diseases from diverse perspectives, which contain the multi-order proximity information from different views. These features for miRNAs of *S* views and diseases of *Q* views can be expressed as:

6 $$\begin{aligned}{} & {} \left\{ \left\{ X_1^{(1)}, X_2^{(1)}, \cdots , X_K^{(1)}\right\} , \cdots ,\left\{ X_1^{(S)}, X_2^{(S)}, \cdots , X_K^{(S)}\right\} \right\} , \end{aligned}$$

7 $$\begin{aligned}{} & {} \quad \left\{ \left\{ Y_1^{(1)}, Y_2^{(1)}, \cdots , Y_K^{(1)}\right\} , \cdots ,\left\{ Y_1^{(Q)}, Y_2^{(Q)}, \cdots , Y_K^{(Q)}\right\} \right\} . \end{aligned}$$

#### Multi-order proximity fusion by CNN

For miRNAs and diseases, multiple feature matrices from different views can be regarded as multiple channels of an image. To the best of our knowledge, CNN utilizes convolutional filters to generate feature maps, which has made a huge breakthrough in computer vision. Therefore, we use CNN to further extract features and obtain high-order proximity representations of miRNA and disease. Given miRNA channel embedding $X_m=\left[ x_1, x_2, \cdots , x_{C_m^{in}}\right]$, the final high-order proximity representation $\widehat{X}_m$ is calculated as follows:

8 $$\begin{aligned} \begin{aligned} output_t=\sum _{i=1}^{C_m^{i n}} x_i \otimes W_m{ }^t+b_m{ }^t, \end{aligned} \end{aligned}$$

where $C_m^{in}=S \times K$, $\otimes$ represents the convolution operator, and $W_m{ }^t$ and $b_m{ }^t$ are the *t*-th convolution filter and bias vector respectively. $output_t$ is the representation from the *t*-th output channel, where $t=1,2, \cdots , C_{out}$ and $C_{out}$ is the number of CNN filters. By stacking the representations from all output channels, the final high-order proximity representation of miRNA $\hat{X}_m \in R^{n m * C_{out}}$ is obtained. Similarly, the disease high-order proximity representation $\widehat{Y}_d$ can be got.

In order to preserve the local and global structural features in the similarity network views, we respectively combine the first-order and high-order representations of miRNA and disease as their structure embeddings, which can be defined as:

9 $$\begin{aligned}{} & {} \tilde{X}_m = G_m^{(1)} \left\| G_m^{(2)} \right\| \hat{X}_m, \end{aligned}$$

10 $$\begin{aligned}{} & {} \quad \tilde{Y}_d = G_d^{(1)} \left\| G_d^{(2)}\right\| \hat{Y}_d, \end{aligned}$$

where $\Vert$ denotes the concatenation operation, and $\tilde{X}_m$ and $\tilde{Y}_d$ are respectively the embeddings of miRNA and disease obtained from similarity views.

### Attention network with neural aggregation

GAT [38] is a powerful graph neural network with good performance in processing graph-structured data. GAT learns the embedding of the central node by assigning different weights to distinct neighbors, which aggregates the information of the neighbors to generate a useful representation of the central node. Therefore, inspired by [38, 49], to achieve the effective fusion of heterogeneous information, we design an attention network with neural aggregation to aggregate the structural features (that are, $\tilde{X}_m$ and $\tilde{Y}_d$, which include direct and indirect neighbor information) from nodes of different types based on the MDA network. Specifically, firstly, since different kinds of nodes have different feature spaces, we use node-type transformation matrices to map them to the same feature space, which is expressed as follows:

11 $$\begin{aligned} \begin{aligned} h^{(l)}=\left[ \begin{array}{l}\tilde{X}_m W_m^{(l)} \\ \tilde{Y}_d W_d^{(l)} \end{array}\right] , \end{aligned} \end{aligned}$$

where $h^{(l)} \in R^{(n m+n d) * f_{tran}}$ is the transformed high-level features in the *l*-th attention head, and $W_m^{(l)}$ and $W_d^{(l)}$ denote the transformation matrices of miRNA nodes and disease nodes, respectively. $f_{tran}$ is the feature dimension of miRNA and disease nodes after being transformed.

Furthermore, we use an attention mechanism to learn the importance of neighbor nodes of each node, and then fuse the representations of neighbor nodes according to the attention score to enhance the representation of the center node. For example, the attention score $e_{i j}^{(l)}$ between miRNA *i* and disease *j* can be defined as:

12 $$\begin{aligned} \begin{aligned} e_{i j}^{(l)}={\text {LeakyReLU}}\left( \left( h_i^{(l)}\right) \left( h_j^{(l)}\right)^T \right) , \end{aligned} \end{aligned}$$

where $h_i^{(l)}$ and $h_j^{(l)}$ are respectively the transformed features of miRNA *i* and disease *j*, and ${\text {LeakyReLU}}$ is the activation function. Then, we obtain the attention coefficient $a_{i j}^{(l)}$ by applying the ${\text {softmax}}$ function to normalize the attention score, which is shown as follows:

13 $$\begin{aligned} \begin{aligned} a_{i j}^{(l)}={\text {softmax}}\left( e_{i j}^{(l)}\right) =\frac{\exp \left( e_{i j}^{(l)}\right) }{\sum _{t \in \mathcal {N}_i} \exp \left( e_{i t}^{(l)}\right) }, \end{aligned} \end{aligned}$$

where $\mathcal {N}_i$ denotes all neighbors of miRNA *i* in the adjacent matrix $G_A$. After obtaining the importance of each neighbor node to the central node *i*, we can obtain the heterogeneous neighbor representation of miRNA *i* by integrating neighbor features according to the attention coefficient:

14 $$\begin{aligned} \begin{aligned} h_{\mathcal {N}_i}^{(l)}=\sigma \left( \sum _{t \in \mathcal {N}_i} a_{i t}^{(l)} h_t^{(l)}\right) , \end{aligned} \end{aligned}$$

where $\sigma$ represents the nonlinear activation function. Similarly, we can get the heterogeneous neighbor representations of disease nodes.

Since $h_{\mathcal {N}}^{(l)}$ only integrates the representations of heterogeneous neighbor nodes and neglects the representation of the node itself, we design a neural aggregator to integrate the node representation $h^{(l)}$ and its heterogeneous neighbor representation $h_{\mathcal {N}}^{(l)}$, which facilitates the information interaction between a node and its heterogeneous neighbors by using the fully connected layers (FCLs). And the enhanced feature $Z^{(l)}$ is represented as follows:

15 $$\begin{aligned} \begin{aligned} Z^{(l)}=\left( \left( h^{(l)} \Vert h_{\mathcal {N}}^{(l)}\right)W_1^{(l)} +b_1^{(l)}\right) \Vert \left( \left( h^{(l)}+h_{\mathcal {N}}^{(l)}\right)W_2^{(l)} +b_2^{(l)}\right) , \end{aligned} \end{aligned}$$

where $W_1^{(l)}$, $W_2^{(l)}$, $b_1^{(l)}$ and $b_2^{(l)}$ are respectively trainable weight and bias matrices.

Finally, similar to standard GAT [38], we use the following multi-head mechanism to obtain the final embedding of miRNA and disease:

16 $$\begin{aligned} \begin{aligned} \tilde{Z}=\left[ \begin{array}{c} \tilde{Z}_m \\ \tilde{Z}_d \end{array}\right] ={\parallel }_{l=1}^{L}Z^{\left( l \right) }, \end{aligned} \end{aligned}$$

where *L* is the number of independent attentional heads.

### Association prediction for miRNAs and diseases

According to the obtained feature representation $\tilde{Z}_m$ for miRNAs and feature representation $\tilde{Z}_d$ for diseases, we simply use their inner product to predict the probability scores of associations between miRNAs and diseases, which is described as follows:

17 $$\begin{aligned} \begin{aligned} \hat{A}={\text {sigmoid}}\left( \tilde{Z}_m \tilde{Z}_d{}^T\right) , \end{aligned} \end{aligned}$$

where the larger the value of $\hat{A}_{i j}$, the more likely miRNA *i* is associated with disease *j*, and conversely, the less likely miRNA *i* is related to disease *j*.

Finally, we optimize the parameters of the PATMDA model by minimizing the cross-entropy loss between true labels and predicted values, where the loss function is defined as follows:

18 $$\begin{aligned} \begin{aligned} {\text {loss}}\left( \hat{A}_{i j}, A_{i j}\right) =-\left( A_{i j} \log \hat{A}_{i j}+\left( 1-A_{i j}\right) \log \left( 1-\hat{A}_{i j}\right) \right) , \end{aligned} \end{aligned}$$

where $A_{i j}$ denotes the true label of the association between miRNA *i* and disease *j*, and Adam optimizer [50] is utilized to train the model.

## Data Availability

The datasets that support the findings of this study are available in https://github.com/xxpaaa/PATMDA.

## Abbreviations

miRNA: microRNA
PPMI: Positive point-wise mutual information
MDA: miRNA-disease association
RWR: Random walk with restart
GIP: Gaussian interaction profile
CNN: Convolutional neural network
GAT: Graph attention network
FCL: Fully connected layer
5-CV: 5-Fold cross-validation
AUC: Area under the receiver operating characteristic (ROC) curve
AUPR: Area under the precision/recall (P–R) curve
